# Exploring the feasibility of EEG for pre-hospital detection of medium and large vessel occlusion strokes: a proof-of-concept study

**DOI:** 10.3389/fneur.2025.1509443

**Published:** 2025-03-27

**Authors:** William Peterson, Nithya Ramakrishnan, David Tinklepaugh, Adrian Hamburger, Arthur Kowell, Krag Browder, Nerses Sanossian, Peggy Nguyen, Ezekiel Fink

**Affiliations:** ^1^Asterion AI, Dallas, TX, United States; ^2^Department of Neurology, UCLA Health, Los Angeles, CA, United States; ^3^Roxanna Todd Hodges Stroke Program, University of Southern California, Los Angeles, CA, United States; ^4^Department of Neurology, Keck School of Medicine of the University of Southern California, Los Angeles, CA, United States

**Keywords:** EEG, stroke, emergency care, prehospital / EMS, large vessel occlusion

## Abstract

**Introduction:**

Early and accurate identification of stroke subtypes, particularly medium (MeVO) and large vessel occlusions (LVO), is critical for timely intervention and improving patient outcomes. Current pre-hospital diagnostic methods are limited in sensitivity, delaying treatment for ischemic stroke candidates eligible for endovascular thrombectomy (EVT).

**Methods:**

This proof-of-concept study explores the feasibility of using electroencephalography (EEG) as a diagnostic tool for pre-hospital detection of MeVO and LVO strokes. Conducted in the emergency department setting, this study assessed the efficacy of quantitative EEG biomarkers in differentiating MeVO/LVO-positive cases (*n* = 4) from MeVO/LVO-negative cases (*n* = 23). EEG data was acquired using both dry and wet electrode systems, with wet electrodes yielding lower attrition rates arising from superior signal quality.

**Results:**

Findings from MeVO- and LVO-positive subjects revealed hemispheric asymmetry in delta and alpha frequency bands, particularly in frontal and temporal regions, as well as a global attenuation of power irrespective of the region of stroke.

**Discussion:**

This study supports the potential of EEG for real-time, non-invasive stroke detection in pre-hospital and clinical environments, demonstrating the need for wet EEG systems for reliable signal acquisition. Future work aims to validate the use of EEG in the pre-hospital setting in an effort to facilitate rapid triage and reduce time to treatment for stroke patients.

## 1 Introduction

The prehospital setting plays a pivotal role in the timely diagnosis and management of strokes, necessitating swift and accurate identification to optimize patient outcomes. Recent studies have emphasized the critical importance of early detection outside hospital settings to facilitate rapid triage and appropriate transport to hospitals equipped to administer specialized treatments such as endovascular thrombectomy (EVT). Prehospital identification of stroke patients who are EVT candidates reduces the time to treatment initiation, reducing morbidity and mortality and improving functional outcomes for stroke patients (1). Stroke centers with the ability to administer EVT are also equipped with advanced imaging modalities, including computed tomography (CT) and magnetic resonance imaging (MRI), to aid in the accurate selection of cases for EVT (2). Accurate and effective stroke diagnosis in prehospital emergency care can enhance treatment efficacy and reduce long-term disability.

Endovascular thrombectomy (EVT) is a minimally invasive surgical procedure designed to remove a blood clot from a blocked artery supplying the brain, thereby restoring blood flow in patients experiencing acute ischemic stroke. EVT targets include intracranial occlusions of M1 and M2 segments of the middle cerebral artery (MCA) and basilar artery occlusions. Benefits of EVT for anterior cerebral artery (ACA) occlusions and more distal MCA occlusions are less clear. Patients presenting with an LVO within 6 h of onset are eligible for EVT. For those presenting between 6- and 24-h post-onset, imaging is required to identify a perfusion-diffusion mismatch, suggesting a salvageable penumbra and benefit from EVT. These criteria are highlighted in the DAWN trial by Nogueira et al. (3), which demonstrated that patients selected based on clinical and imaging criteria benefited significantly from EVT, emphasizing the importance of timely and accurate patient selection for this procedure. Recent literature has discussed the inclusion of medium vessel occlusion (MeVO) strokes of the anterior cerebral artery, middle cerebral artery, and posterior cerebral artery for endovascular thrombectomy, with at least two ongoing clinical trials exploring its benefits (DISTAL and ESCAPE-MeVO). Improved identification of LVO and MeVO strokes is a growing need for the proper triage of stroke patients in the prehospital setting.

Electroencephalography (EEG), which is traditionally used to diagnose seizures and manage epilepsy, holds significant potential for pre-hospital detection of MeVO/LVO stroke due to its ability to detect changes in brain electrical activity that precedes visible changes in imaging studies. For example, certain EEG patterns, such as focal slowing or asymmetry, can suggest an area of reduced blood flow. EEG has shown utility in monitoring cerebral ischemia, especially during intraoperative procedures, and more recently, detecting acute ischemic stroke (4, 5). EEG is being developed for pre-hospital MeVO/LVO detection independently, and currently administered prehospital stroke scales such as the Los Angeles Prehospital Stroke Scale and Cincinnati Prehospital Stroke Scale remain the current standard for prehospital stroke triage.

For EEG to be effective in pre-hospital stroke detection, recording quality is critical and influenced by several factors, most notably the type of electrode utilized for signal acquisition. Both wet and dry electrode options exist, each with distinct advantages and challenges. Wet electrodes utilize a conductive gel or saline solution to reduce skin impedance that improves the signal to noise ratio but requires a longer setup time. Dry electrodes are more rapidly deployed but suffer from a higher impedance and thus are susceptible to more artifacts. This study explored the use of both EEG electrode configurations in the emergency department and found reduced attrition rates with the wet electrode system.

In this article, we provide preliminary results from a proof-of-concept study demonstrating the feasibility of using EEG to distinguish MeVO/LVO stroke in an emergency department setting. Results from this study will help with developing a tool for real-time detection of MeVO/LVO strokes prior to hospital admission to facilitate efficient triage for appropriate medical treatment and improve patient outcomes.

## 2 Materials and methods

### 2.1 Study design

This was a single-center investigator-initiated study conducted at the Los Angeles County General Medical Center, approved through the Institutional Review Board. Adult patients in the emergency department, diagnosed with likely acute ischemic stroke were enrolled in this study while they received acute stroke care. After the initial assessment that took place in the emergency department, which includes obtaining a history, a clinical examination, EKG, bloodwork, and brain imaging, patients diagnosed with a stroke were evaluated for treatment with intravenous thrombolysis and in cases of MeVO/LVO, for endovascular therapy. Once the treatment plan was determined, patients in the emergency department underwent EEG recordings of 5–10 min duration.

### 2.2 Inclusion and exclusion criteria

Adult patients had to be 18 years or older, have a diagnosis or suspicion of acute stroke, with symptom onset less than 6 h and provide a concern for MeVO/LVO by the treating team. All patients were identified from stroke team activations from LAC+USC Medical Center. Patients were excluded from the study if they were unable to provide informed consent, had a severe respiratory disease, was in a coma, had a systolic blood pressure of less than 90 or more than 220 or had a pre-existing neurologic, psychiatric or advanced systemic disease that interfered with EEG acquisition.

### 2.3 EEG acquisition and preprocessing

The gtec g.Nautilus PRO with the gtec g.Sahara Hybrid dry or wet electrode configuration (32-channel cap with the 10/20 configuration) was used in this recording. Of the 32 channels, we used 19 channels (FP1, FP2, F3, F4, F7, C3, C4, T3, T4, P3, P4, T5, T6, O1, O2, FZ, CZ, PZ) as they consistently had better contact with the scalp. The g.Sahara Hybrid electrodes allow for EEG acquisition with and without conductive gel (dry and wet). We used the active dry EEG electrodes for the initial data collection, but the configuration with conductive gel provided far better signal quality, attributable to lower impedance levels. The setup time for the dry electrodes only took a couple of minutes, while the wet electrodes took 5–10 min for setup per patient. The resulting dataset of EEG cases were preprocessed using artifact subspace reconstruction (ASR) and independent component analysis (ICA) to mitigate contamination from noise. The cleanest window spanning a minimum duration of 15 s, up to 3-min was selected for further analysis. If a time window spanning greater than 10 s could not be selected, the recording was dropped from the analysis.

Quantitative features were computed from the preprocessed recordings and compared using a Mann–Whitney *U* test, due to its robustness to outliers and ability to handle smaller sample sizes. Power spectrums for both cohorts were calculated using Welch’s method to ensure equivalent spectral resolution independent of varying time window lengths. Topographies of the MeVO and LVO cases were generated to determine in which cases slowing, or general attenuation across all frequency bands, was most consistent with the region of the infarct.

### 2.4 Analytical methods

Quantitative EEG features included in the analysis covered temporal, spectral, and spatial domains. The complete list of features is provided under Table 1.

**Table 1:** Descriptions of features.

Feature set	Description
Spectral ratios	Ratio of the power between slower frequencies (delta and alpha) and faster frequency bands (alpha, beta).
Band power difference	Simple difference in power among frequency bands.
Relative band powers	Average power for each frequency band normalized by sum of all powers.
Brain symmetry index	Quantifies interhemispheric spectral asymmetry; Computed as the mean difference in absolute power between a sensor and its contralateral pair.
Inter-hemispheric amplitude ratio	Quantifies interhemispheric amplitude asymmetry; Revised as the difference in mean amplitude envelopes between a sensor and its contralateral pair.
Sample entropy	Quantifies degree of regularity or predictability for a time series.
Hurst exponent	Quantifies long-term memory of time series by analyzing tendency of a signal to regress to its mean, or conversely, trend in a given direction.

Due to the stroke’s localization to the region of the brain suffering from poor cerebral perfusion, we quantified asymmetries in the EEG according to amplitude and frequency powers. Asymmetry features were z-scored, and the absolute value was applied to remove any directionality from their interpretation. Additionally, we did not limit asymmetry to strictly hemispheric differences but also included longitudinal comparisons of power. Normally, features capturing asymmetries in EEG focus on hemispheric differences and while effective, may miss instances of reduced or increased power to an entire region (for example, FIRDA). Mann Whitney *U* tests allowed us to examine the asymmetry features showing the greatest differences in distribution.

## 3 Results

**Figure 1:**
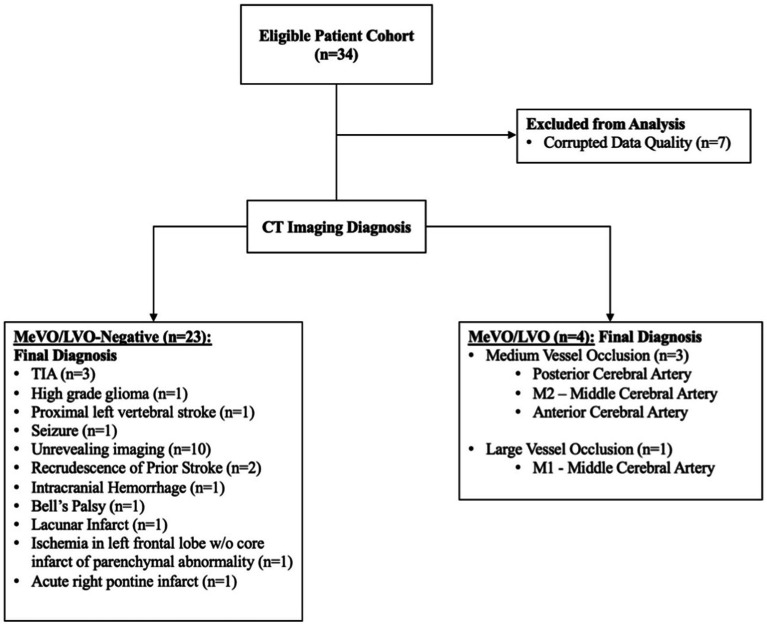
Flowchart of participants and reported diagnosis.

Thirty-four subjects presenting to the emergency department (ED) with suspected stroke symptoms were enrolled in this study. Of the 34 subjects, seven were diagnosed with medium or large vessel occlusions (3 LVO, 4 MeVO). The remaining 27 cases consisted of non-MeVO/LVO, implying an overlap in symptom presentation without an underlying stroke. Seven subjects (2 LVO, 1 MeVO, 4 negative MeVO/LVO) were excluded due to inadequate EEG signal quality. A complete breakdown of the participant pool is provided in the flowchart below (Figure 1).

Patient ages ranged from 24 to 93. The mean National Institute of Health Stroke Scale (NIHSS) score was 3.75 and 3.96 for the MeVO/LVO and MeVO/LVO-negative cohorts, respectively (Table 2).

**Table 2:** Demographics of stroke cohort.

Demographic	MeVO & LVO (*n* = 4)	No MeVO/LVO (*n* = 14)
Age		
Mean (Std. Dev)	55.75 (27)	54.87 (19)
Sex		
Male	2 (50%)	16 (70%)
Female	2 (50%)	7 (30%)
NIHSS Mean (Std. Dev)	3.75 (1.89)	3.96 (2.38)

**Figure 2:**
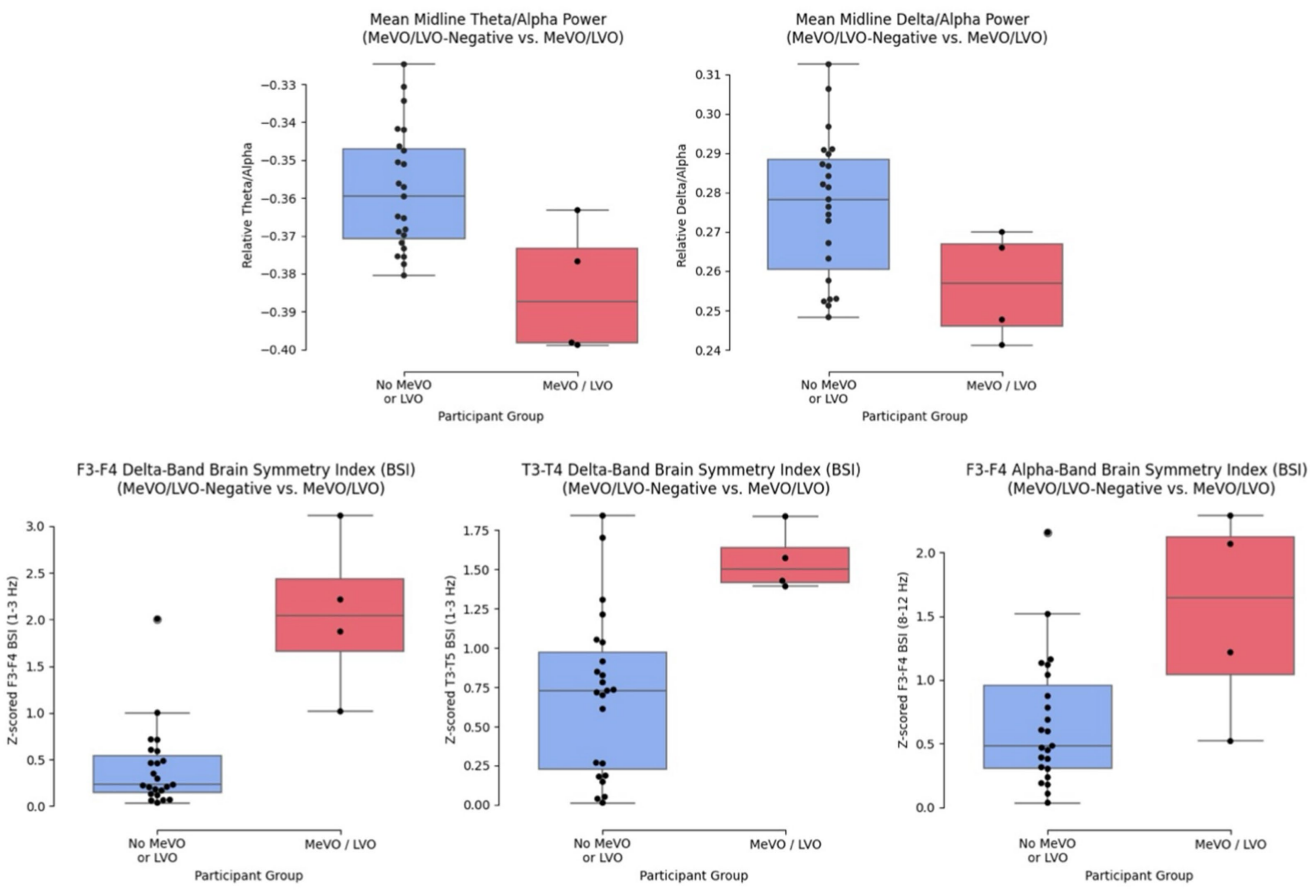
Features displaying greatest significant difference between cohorts.

Quantitative EEG features demonstrated a strong ability to distinguish between the non-MeVO/LVO cohort and the MeVO/LVO group. Figure 2 below shows the Mean midline Theta/Alpha and Delta/Alpha power between the MeVO/LVO group and the MeVO/LVO-negative group as well as the Delta and Alpha band brain symmetry index, computed as the mean power difference between a sensor and contralateral pair in the frontal (F3-F4) and temporal (T3-T4) electrodes between the two groups. Our results demonstrate greatest asymmetries in the delta and alpha bands, primarily in the frontal and temporal positioned channels.

**Figure 3:**
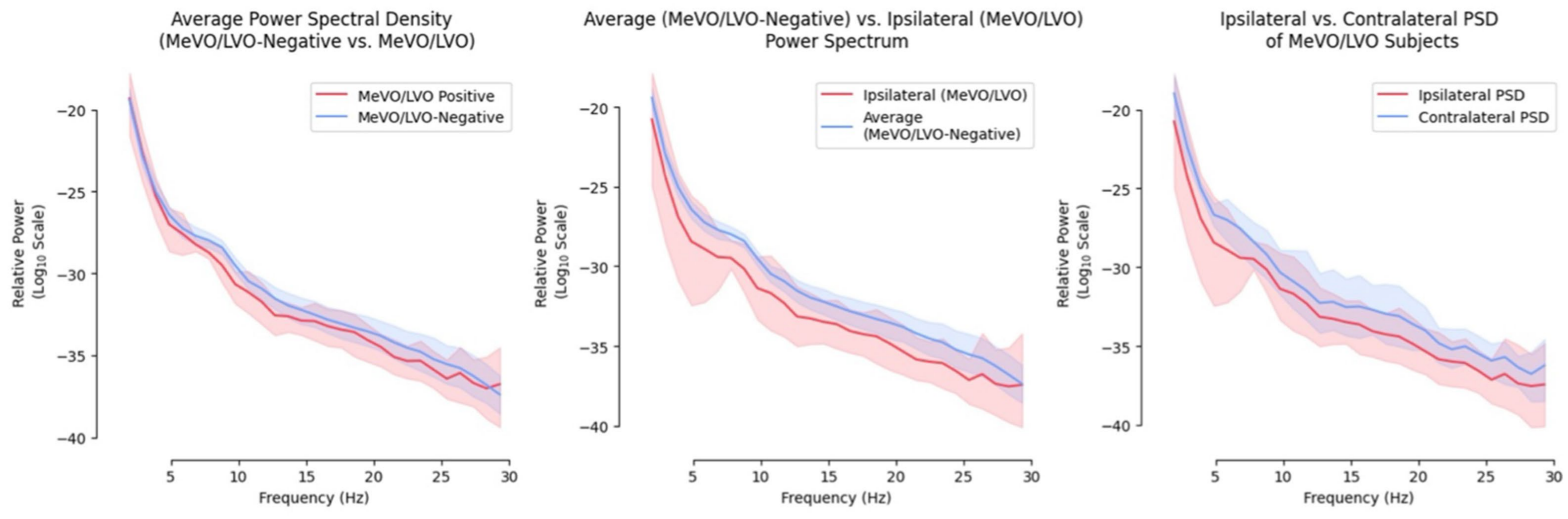
Comparison of power spectrums between both participant groups.

Examining the power spectrums between the two participant groups demonstrates the attenuation of power seen across all frequency bands (Figure 3). Most notably, the ipsilateral power spectrum demonstrated the strongest attenuation relative to both the contralateral and non-stroke group’s power.

**Figure 4:**
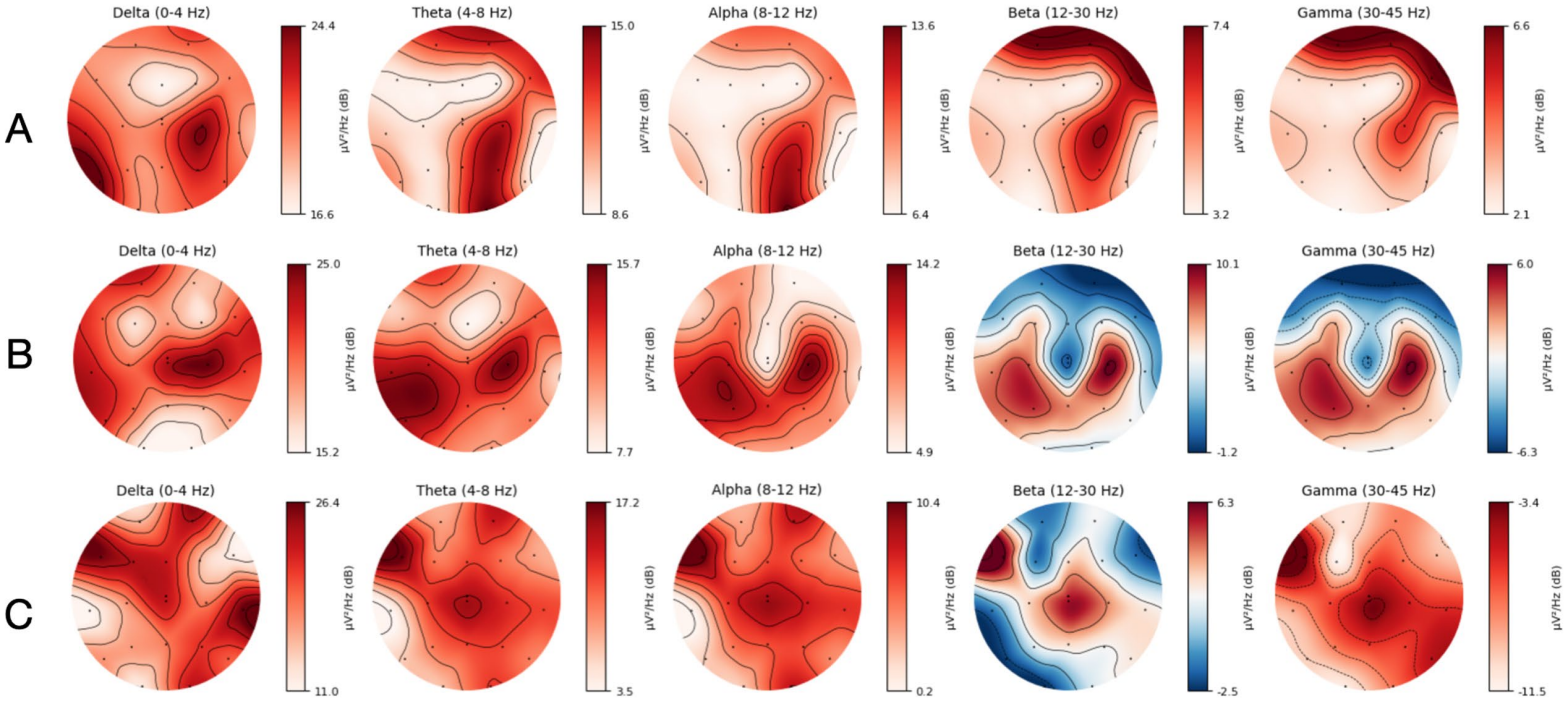
**(A)** Topographical map displaying spatial distribution of frequency band powers for a patient with a right Middle Cerebral Artery (MCA) MeVO. **(B)** Topographical map displaying spatial distribution of frequency band powers for a patient with a right Anterior Cerebral Artery (ACA) MeVO. **(C)** Topographical map displaying spatial distribution of frequency band powers for a patient with a left Middle Cerebral Artery (MCA) LVO.

**Figure 5:**
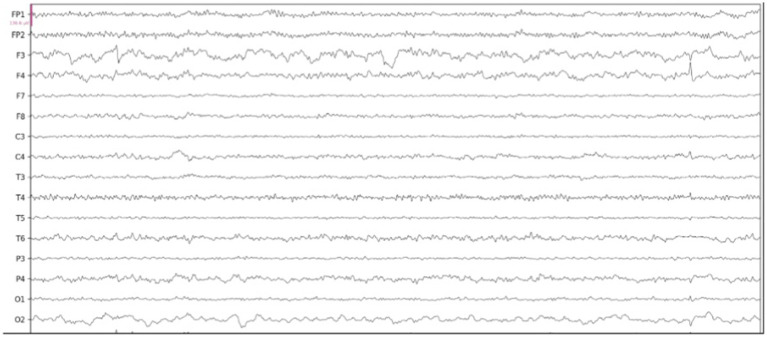
Electroencephalogram (EEG) recording from a patient presenting with left PCA MeVO.

**Figure 6:**
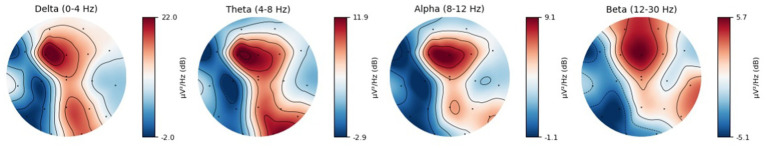
Topographical power plot from a patient presenting with left PCA MeVO.

A general attenuation in power, most predominantly observed in the faster frequency bands, was consistent with the region of infarct in three out of four of the MeVO/LVO patients (Figure 4), confirmed by a manual review of topographical power plots. The EEG and topographical plot from a patient presenting with a medium vessel occlusion located in the left posterior cerebral artery is presented below. The recording and topographical plots demonstrate strong attenuation at the site of the stroke, affecting the left temporal and occipital lobes (Figure 5; channels: C3, T3, T5, P3, O1). This pattern of reduced power is seen across all frequency bands (Figure 6). Contralateral to the site of occlusion, there is evidence of slowing, particularly in the occipital region (channel: O2). This presentation of contralateral slowing and focal attenuation may indicate generalized cerebral dysfunction secondary to the stroke.

## 4 Discussion

EEG offers a low-profile, easy-to-use solution for detecting cerebral dysfunction related to underlying infarctions and can be quickly deployed to prehospital and clinical settings. Its adoption in prehospital environments has been delayed due to contamination from environmental noise and difficulties related to hardware access. EEG deployment to the prehospital setting has further practical limitations, including its inability to be quickly interpreted in the absence of trained neurologists and epileptologists. The use of quantitative EEG features bypasses this obstacle in interpretation and provides a simplified visualization of the recording. Our findings support the use of these quantitative features in the delineation of MeVO and LVO cases from non-MeVO/LVO, demonstrating their potential to be deployed to prehospital and clinical environments for fast, point-of-care diagnostic testing. In this feasibility study, we present a case for the use of EEG in recognizing signs of stroke in the emergency department, presenting quantitative EEG features with strong discriminatory power. We also demonstrate the impracticality of dry EEG in the emergency department, where environmental noise dominates the signal, highlighting the need for an active, wet system that can mitigate signal interference.

### 4.1 EEG acquisition optimization

In this study, we initially aimed to utilize dry EEG in the emergency department to leverage the faster setup time and evaluate its utility in distinguishing MeVO/LVO strokes from non-MeVO/LVO in an emergency department. The decision to utilize a dry vs. wet EEG configuration (with and without a conductive medium) depends on several factors, including the quality of the EEG system, the ease of application and training for new users and the environmental noise that contributes to the signal quality. In the literature, there is better support for the use of wet EEG as it provides a more robust and noise-reduced signal (6). All seven recordings excluded from the study due to unacceptably high noise-to-signal ratio were obtained with dry EEG electrodes, confirming the superiority of wet EEG electrodes utilizing conductive gel. While offering a quicker setup time, the tradeoff for a dry configuration is seemingly not worthwhile if signal quality disrupts the functionality of EEG biomarkers for identifying medium and large vessel occlusions. Through the use of artifact removal algorithms, we demonstrate environmental noise can be mitigated enough to enrich the computation of quantitative EEG feature values.

### 4.2 Discriminatory power of quantitative EEG biomarkers

Elevated brain symmetry index (BSI) values indicate significant asymmetry, often correlating with the severity of the stroke and the extent of functional impairment. Research has demonstrated that stroke patients typically exhibit higher BSI values compared to healthy individuals, reflecting the disrupted neural activity in the affected hemisphere. Studies by van Putten and Tavy (7) and Sheorajpanday et al. (8) have shown that BSI can serve as a reliable biomarker for stroke severity and prognosis. In our participant pool of four stroke subjects (Figure 2), asymmetrical differences were concentrated in the frontal and temporal regions; however, we expect that a larger stroke cohort, composed of a more diverse set of stroke cases spanning various cerebral arteries, would result in more channels displaying signs of asymmetry.

An elevated delta/alpha ratio (DAR), characterized by increased delta power and decreased alpha power, or an elevated theta/alpha ratio (TAR) typically indicates severe cortical dysfunction and poor prognosis. Delta and theta waves are prevalent in regions with impaired neuronal activity, while alpha waves are linked to relaxed, wakeful states and efficient cognitive processing. Thus, a higher DAR/TAR suggests greater disruption in normal brain function. Specifically, increased slow wave activity and decreased alpha band activity are commonly observed in the stroke-affected hemisphere, reflecting disruptions in normal brain function and connectivity (9, 10). Interestingly, while an increase in DAR/TAR is normally seen in patients suffering from ischemia, we observed a general decrease of DAR/TAR in the stroke cohort relative to the MeVO/LVO-negative cases (Figure 2). We hypothesize this is less the result of increased slow wave activity (delta and theta rhythms) and suppressed alpha activity in the non-stroke group but related to an attenuation of power across all frequency bands in the stroke cohort. This implies that the observed attenuation in the stroke cohort was not confined to the faster frequencies but also attenuated the slower delta and theta bands. This reduction in spectral power reflects diminished neural activity and connectivity in the affected brain regions.

In Figure 3, we demonstrate a global attenuation of power in all frequencies for the MeVo/LVO group, with the strongest attenuation located on the ipsilateral brain regions compared to contralateral brain regions. This could be due to the ischemic abnormality occurring in one hemisphere and not both. A study of EEG in 48 patients with acute ischemic stroke (11) revealed a distinctive EEG pattern of regional amplitude attenuation of all frequencies without supervening delta in the ischemic hemisphere. This measure called RAWOD was found to identify massive ischemic stroke earlier than CT or MRI (12).

In Figures 5, 6, we present results from a single patient with left hemisphere posterior cerebral artery stroke (PCA) MeVO, where we found a pattern of reduced power in the alpha and beta bands. Consistent with our findings, several EEG studies in acute and subacute stroke found a relationship between reduced cerebral blood flow (CBF) and increased alpha and beta power. For example, a recent magnetoencephalography (MEG) study found a significant positive correlation between CBF and alpha and beta power, indicating that reduction in blood flow tends to be associated with attenuation of higher frequency oscillations in alpha and beta range (13).

### 4.3 Automated near-real time analysis of EEG for detection of MeVO/LVO

EEG is a promising tool for real-time detection of MeVO/LVO stroke due to its ability to monitor brain activity non-invasively, its high temporal resolution, and its sensitivity to changes in cortical function. Stroke often leads to immediate and distinct alterations in brain oscillations, often correlated with changes to CBF and occurring on the same hemisphere as the ischemic or hemorrhagic abnormality. These EEG changes are found earlier than CT or MRI or when there are no abnormal signs in the initial CT scan (14, 15), making it a highly sensitive tool that can be deployed in a pre-hospital or ambulatory setting, making it suitable for use in emergency settings where timely diagnosis is critical.

### 4.4 Limitations

While the time required for EEG acquisition, processing, and classification was explicitly studied in the context of prehospital workflow in this study, it did provide insights on the complexity of real-time implementation which optimization on multiple aspects to ensure rapid decision-making in time-sensitive stroke care. One of the primary challenges we faced was in the processing of EEG signals. While we used objective, scientifically robust methods such as artifact subspace reconstruction (ASR) and independent component analysis (ICA), there was still a fair amount of manual selection of a clean EEG time window and removal of artifactual components. This approach is time-intensive and not practical in prehospital settings, where rapid, automated processing is essential. Future work should focus on developing robust algorithms for real-time, fully automated analysis. Another significant limitation in this study was the highly artifactual data we obtained using the dry EEG system. While we favored the dry EEG electrodes for its quick application in a pre-hospital setting, we found that there was an increased likelihood of signal artifacts, partly due to the environmental noise from the ER environment that caused some of the MeVO/LVO patients to be excluded from the analysis, thus reducing our sample size further. Future work should attempt enhanced signal processing techniques to be able to utilize rapid-application dry EEG systems for this purpose. Due to the nature of this article being a proof-of-concept study, the sample size was limited, which may reduce the generalizability of the findings. Larger, more diverse populations are necessary to confirm the utility of EEG in prehospital settings for detecting large vessel occlusion (LVO) strokes.

## 5 Conclusion

This study aimed to assess the feasibility of EEG in the emergency department for identifying patients with confirmed MeVO/LVO prior to hospital admission. We evaluated literature-supported EEG biomarkers and found strong discriminatory power in distinguishing MeVO/LVO cases. We further demonstrate the dominance of wet EEG over a dry configuration, with the wet EEG leading to significantly lower attrition rates. Overall, this work validates the use of wet EEG as a rapid, low-profile solution to facilitate efficient diagnosis and triage of patients with medium and large vessel occlusions prior to hospital admission.

## Data Availability

The datasets presented in this article are not readily available because ethical and privacy restrictions. The figures included in this article will be available upon request. Requests to access the datasets should be directed to EFink@hspmgroup.com.
